# France Decreasing in Population

**Published:** 1889-11

**Authors:** 


FRANCE DECRASING IN POPULATION.—It is stated in the British Med. Jour. that the population of France is decreasing. There is diminution of marriages, augmentative of divorces, an increase of illegitimate and a decrease of legitimate births. This is an unfortunate record for any country, and evinces a demoralization of the masses which gives a bad outlook for the future of France.

